# Landscape Suitability in Botswana for the Conservation of Its Six Large African Carnivores

**DOI:** 10.1371/journal.pone.0100202

**Published:** 2014-06-20

**Authors:** Hanlie E. K. Winterbach, Christiaan W. Winterbach, Michael J. Somers

**Affiliations:** 1 Tau Consultants (Pty) Ltd, Maun, Botswana; 2 Centre for Wildlife Management, University of Pretoria, Pretoria, South Africa; 3 Centre for Invasion Biology, University of Pretoria, Pretoria, South Africa; Università degli Studi di Napoli Federico II, Italy

## Abstract

Wide-ranging large carnivores often range beyond the boundaries of protected areas into human-dominated areas. Mapping out potentially suitable habitats on a country-wide scale and identifying areas with potentially high levels of threats to large carnivore survival is necessary to develop national conservation action plans. We used a novel approach to map and identify these areas in Botswana for its large carnivore guild consisting of lion (*Panthera leo*), leopard (*Panthera pardus*), spotted hyaena (*Crocuta crocuta*), brown hyaena (*Hyaena brunnea*), cheetah (*Acinonyx jubatus*) and African wild dog (*Lycaon pictus*). The habitat suitability for large carnivores depends primarily on prey availability, interspecific competition, and conflict with humans. Prey availability is most likely the strongest natural determinant. We used the distribution of biomass of typical wild ungulate species occurring in Botswana which is preyed upon by the six large carnivores to evaluate the potential suitability of the different management zones in the country to sustain large carnivore populations. In areas where a high biomass of large prey species occurred, we assumed interspecific competition between dominant and subordinated competitors to be high. This reduced the suitability of these areas for conservation of subordinate competitors, and vice versa. We used the percentage of prey biomass of the total prey and livestock biomass to identify areas with potentially high levels of conflict in agricultural areas. High to medium biomass of large prey was mostly confined to conservation zones, while small prey biomass was more evenly spread across large parts of the country. This necessitates different conservation strategies for carnivores with a preference for large prey, and those that can persist in the agricultural areas. To ensure connectivity between populations inside Botswana and also with its neighbours, a number of critical areas for priority management actions exist in the agricultural zones.

## Introduction

World-wide few protected areas still exist that are large enough to contain wide-ranging large carnivores. In addition, subordinate species such as cheetahs (*Acinonyx jubatus*) and African wild dogs (*Lycaon pictus*) tend to move into areas with low densities of their dominant competitors which frequently fall inside agricultural areas. This necessitates a conservation action plan on a country-wide scale covering both conservation and human-dominated areas. Understanding the dynamics of large carnivore distribution and factors threatening their survival on a national level is thus crucial for developing effective management strategies, especially in critical areas required for the maintenance of healthy viable populations.

The three main factors that influence the distribution and population viability of large carnivores are prey availability, interspecific competition, and conflict with humans [1]. Each large carnivore species has its preferred prey species and prey in preferred weight ranges [2], and the availability of prey most likely plays the primary role in determining the suitability of an area for persistence [2]–[4]. However, in areas where natural prey is scarce, predators often resort to killing livestock which is the most widespread cause of conflict with people [5]. In Botswana, wild prey and livestock occur widespread across the country. Thus local prey availability and numbers of livestock can serve as indicators of suitable habitats for the different large carnivore species and levels of potential human-carnivore conflict [1].

Botswana plays a vital role in the conservation of six of the seven large African carnivores. It is home to the second largest lion (*Panthera leo*) population [6], one of the three largest remaining populations of the endangered African wild dog [7], the second largest population of cheetahs [8], and one of the two largest populations of brown hyaenas (*Hyaena brunnea*). It is also a core country for one of the five largest transboundary lion populations [9], the largest transboundary African wild dog population, and the largest known resident population of cheetahs in southern Africa [10].

Here we assess the suitability of Botswana on a landscape level to conserve its large carnivore guild. We used a novel approach by mapping wild prey biomass as an indicator of the potential of areas to sustain large carnivores, and the percentage prey of the total available biomass (wild prey and livestock) as an indicator of conflict with humans. We provide a landscape map of suitable conservation areas and critical areas for priority management actions.

## Background on Botswana

The Republic of Botswana covers an area of approximately 582,000 km^2^ and shares borders with Namibia, South Africa, Zimbabwe and Zambia. Its human population is around 2 million people (3.5 people/km^2^) with an annual growth rate of 1.9% [11]. The main economies in the country are mining, wildlife tourism, and agriculture [12], but roughly 50% of Botswana's population lives in rural villages and small settlements (cattle posts).

The climate is arid to semi-arid. Mean annual rainfall is spatially and temporally extremely variable, but generally varies from 650 mm in the north-east to 250 mm in the south-west with periodic severe droughts. Average maximum daily temperatures range from 22°C in July to 33°C in January and average minimum temperatures from 5°C to 19°C respectively [13].

Surface water is scarce in Botswana for much of the year. The only two perennial rivers are the Okavango River that fans out into the Okavango Delta, a RAMSAR site that covers some 15,000 km^2^, and the Kwando/Linyanti/Chobe river system that forms the boundary with Namibia and Zambia. South of the Delta lies the Makgadikgadi Pans, which is a seasonal wetland characterised by vast flat, salty depressions. In the Makgadikgadi National Park, natural perennial water holes in the Boteti River are critical dry season water sources for wildlife. Other conservation areas have limited boreholes for wildlife. In the rest of the country, a network of scattered pans and ancient riverbeds may hold water during the wet season. Rural Botswana depends mostly on groundwater accessed through boreholes, whilst the urban areas depend on surface water harvesting [14].

The topography is predominantly flat with a mean altitude above sea level of 1,000 m (515 - 1,491 m a.s.l.). Eighty percent of the country is covered by Kalahari sandveld with nutrient poor sandy soils. Its vegetation ranges from Miombo and mopane (*Colophospermum mopane*) dominated woodland and close-tree Acacia savannah in the north, to more arid and open low tree and shrub savannah with perennial and annual grasses in the south and west. In the pans and riverbeds, nutrient rich soils bear better quality grasses and provide mineral licks for wildlife [15]. The Hardveld in the east consists of rocky hill ranges, more fertile soils, savannah grasslands and - woodlands, and some forest.

Approximately 38% of the country's area is reserved for wildlife conservation: 17% are national parks and game reserves and 21% as Wildlife Management Areas (WMAs). The primary land use in WMAs is wildlife utilization with the aim to protect key areas from incursion by livestock and to develop wildlife in the rural economy. In northern, central and south-western Botswana wildlife is an important economic resource through ecotourism [16], and in the west for game farming. In the south and south-west wildlife numbers have declined drastically with the expansion of human settlements and the increase of livestock numbers, made possible by the provision of boreholes. In addition, Botswana is criss-crossed with non-predator proof veterinary cordon fences to control livestock disease, particularly foot-and-mouth disease [17]. These fences obstruct natural ungulate migratory routes [17], [18]. Due to the variable rainfall regime, there are considerable seasonal variations in the distribution, population and group sizes of ungulate species. Their large and small scale movements, together with the blocking of routes by veterinary fences often lead to ungulate die-offs during drought years [19], [20]. The importance of natural migration routes is evident in other areas of the country; seasonal mass migrations of Burchell's zebra (*Equus burchelli*) and blue wildebeest (*Connochaetes taurinus*) still occur inside the Makgadikgadi National Park [21], and the zebra migration between the Park and the Okavango Delta is the second longest in Africa, after the Serengeti-Mara migration [22]. Protected areas and WMA are not predator-proof fenced, with the exception along the western and southern boundary of the Makgadikgadi National Park, although it provides only a partial barrier due to the poor maintenance of the fence.

Around 57% of Botswana consists of rangeland. Approximately 70% is tribal/communal grazing land, 25% is state land, and 5% is freehold land leased for large-scale commercial ranching [13]. In 2008, Botswana had almost 4,5 million livestock of which 2,2 million were cattle. Approximately 92% of livestock are in the traditional cattle post system on communal grazing land [23]. Livestock (mainly cattle) rearing is the main economic activity over large parts of Botswana and constitutes 70–80% of the agricultural GDP [24]. This is also the largest single source of rural income and plays an important role in the social standing of the local people [25], [26].

Botswana's key environmental issues include depletion of water resources, rangeland degradation and desertification, unsustainable use of veld products and declining numbers of some wildlife species.

## Methods

### Ethics statement

This study was done under the independent research permit no. OP 46/1 LXVIII (133) approved by the Ministry of Environment, Wildlife and Tourism, Private Bag B0199, Gaborone, Botswana.

**Figure 1:**
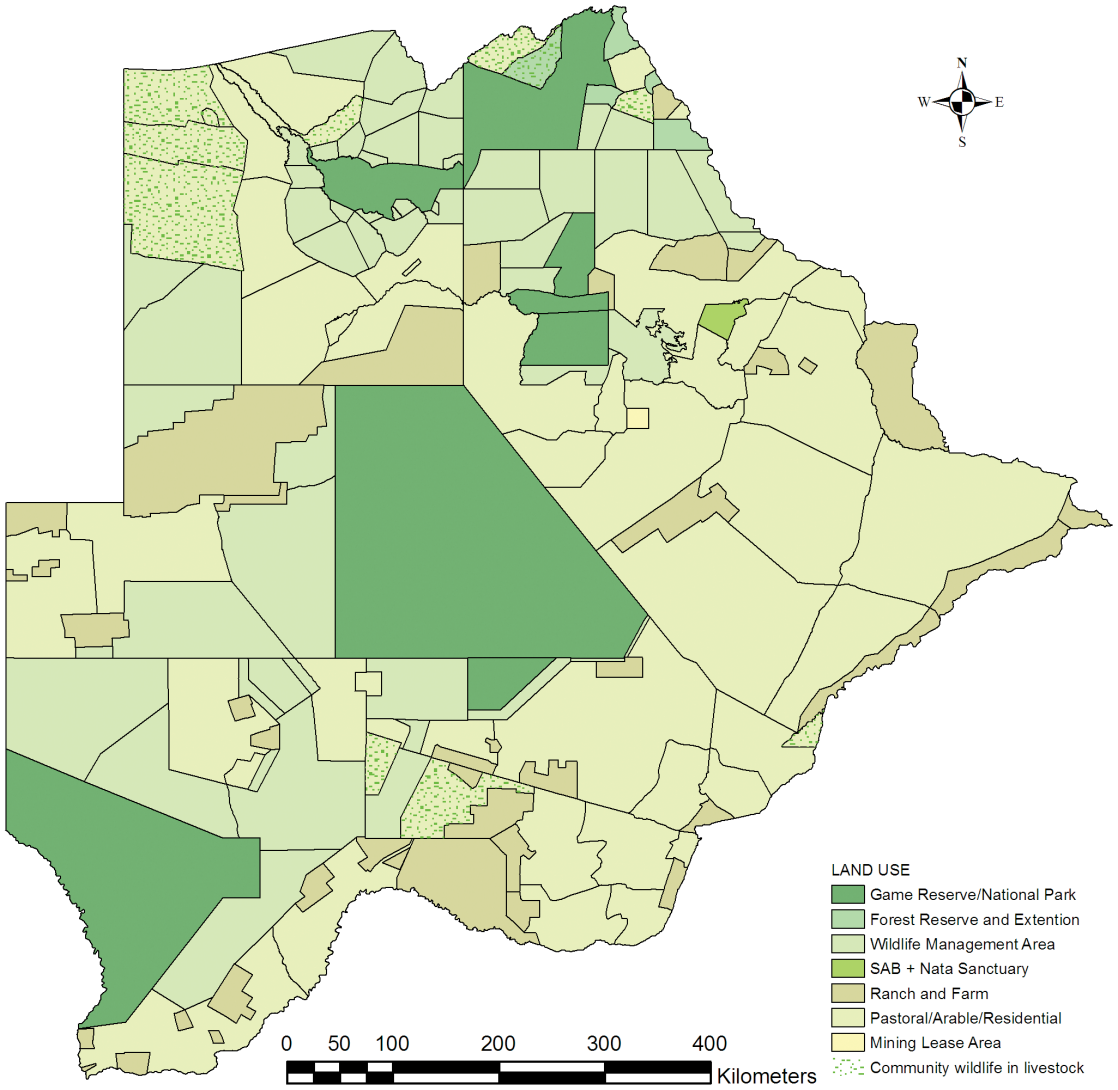
Land use zones in Botswana. Map of the two main land use zones: the Conservation Zones (green colours) consisting of protected areas and Wildlife Management Areas, and the Agricultural Zones (brown colours) consisting of communal grazing land, farms used mainly for livestock production and limited crop production, game ranches, and mining - and residential areas.

Using GIS data layers from the Botswana National Atlas [13], we identified two main management zones, conservation and agricultural. The conservation zones are a combination of protected areas and WMAs. The agricultural zones consist of communal grazing land, farms used mainly for livestock production and limited crop production, game ranches, mining, and residential areas (Figure 1). We obtained wildlife and livestock population numbers from the aerial surveys conducted by the Botswana Department of Wildlife and National Parks which is available in the Botswana Aerial Survey Information System (BASIS) [27]. These survey data are presented in grid cells varying in size according to the stratifications and spacing of the original aerial transects of 1, 3, 6 or 12 minutes [28]. We used the dry season aerial survey data for 2002 and 2003 (which covered the whole country), and 2001, 2004, 2005 and 2007 (which covered the country partially) as it most closely represented the current situation in Botswana. The accuracy of aerial survey counts is influenced by, among others, the visibility – and sighting probability of different animal species, but, overall it tends to undercount most animal species [29]. This is especially true for solitary small cryptic species such as steenbok and duiker. It is, however, the only feasible method to monitor wildlife on a country-wide basis, and within the scope of this study the combined data of six annual dry season aerial surveys was sufficient to determine the broad distribution of wild prey and livestock biomass.

In this paper ‘prey’ refers to wild ungulate species occurring in Botswana that are typical prey for the six large carnivore species, and includes warthog and ostrich. It excludes atypical prey for the large carnivores e.g. elephants (Table 1). For each grid cell we calculated the biomass for large prey, small prey, large livestock, and small livestock. Species classified as large - and small prey are listed in Table 1. Large livestock are cattle (*Bos* spp.), horses (*Equus caballus*) and donkeys (*E. asinus*), and small livestock sheep (*Ovis* spp.) and goats (*Capra hircus*). Biomass was calculated by converting the estimated number of animals per grid cell to Large Stock Units (LSU) using the formula LSU  =  body weight ^0.75^. Body weights of the different species were obtained from BASIS, and species recorded in the survey but not listed in the Table 1 were excluded from the analysis. Mean biomass (LSU/100 km^2^) was calculated per 12′ grid cell (approximately 20.6 km×22.1 km) using the calculated LSU and grid cell sizes of the individual surveys. This data is provided in Spreadsheet S1. The combined aerial surveys covered each grid cell between three and five times. The percentage prey biomass was calculated from the total biomass (prey plus livestock) for large and small species, respectively.

**Table 1:** Typical large African carnivore prey species occurring in Botswana used in the biomass analysis with their corresponding body weight (kg) and Large Stock Unit (LSU) conversion.

Species	Common name	Body weight (kg)	LSU
**Large prey**			
*Giraffa camelopardalis*	Giraffe	750	1.47
*Syncerus caffer*	Buffalo	450	1.00
*Tragelaphus oryx*	Eland	340	0.81
*Hippotragus equinus*	Roan	220	0.58
*Equus burchelli*	Zebra	200	0.54
*Hippotragus niger*	Sable	185	0.51
*Connochaetes taurinus*	Wildebeest	165	0.51
*Oryx gazelle*	Gemsbok	150	0.44
*Tragelaphus strepsiceros*	Greater kudu	136	0.41
*Kobus ellipsiprymnus*	Waterbuck	135	0.41
*Alcelaphus buselaphus*	Hartebeest	125	0.38
*Damaliscus lunatus*	Tsessebe	110	0.35
*Kobus leche*	Lechwe	72	0.25
*Struthio camelus*	Ostrich	68	0.24
**Small prey**			
*Phacochoerus africanus*	Common warthog	45	0.18
*Aepyceros melampus*	Impala	45	0.18
*Redunca* spp.	Common reedbuck	40	0.16
*Antidorcas marsupialis*	Springbok	26	0.12
*Sylvicapra grimmia*	Duiker	15	0.08
*Raphicerus campestris*	Steenbok	10	0.06

Biomass for large and small prey were categorised from marginal to very high (Table 2). The low and high large prey biomass categories were determined using the mean large prey biomass ±1 SD of the 12′ grid cells in reference sites with a low lion density (0.77 animals/100 km^2^) [30] and a high lion density (6–10 animals/100 km^2^) (unpublished data). Values exceeding the mean large prey biomass +1 SD were included in the high biomass category and those between the low and the high categories were used as a medium biomass category. The lowest large prey biomass recorded in an area with resident lions was 0.24–0.26 LSU/km^2^ [31]. We therefore used zero large prey biomass up to 50% of 0.25 LSU/100 km^2^ as the marginal category. The low large prey biomass category fell between marginal and low. Small prey biomass at reference sites with resident cheetah in Ghanzi [32], Jwaneng [33], and Kgalagadi Transfrontier Park [30] were used to categorise high or low small prey biomass. Small - and large livestock were categorized as being present or absent. The categories used for the percentage large prey and percentage small prey were 0, 0>×≤1, 1>×≤5, 5>×≤10 and 10>×≤20.

**Table 2:** Criteria applied to distinguish between the different categories of large – and small prey biomass.

Category	Large prey (LSU/100 km^2^)	Small prey (LSU/100 km^2^)
Marginal	0≥×≤0.125	0
Very low	0.125>×≤0.406	
Low	0.406>×≤1.627	0.000>×≤0.003
Medium	1.627>×≤6.715	0.003>×≤0.0725
High	6.715>×≤16.752	0.0725>
Very High	16.752>	

Observations per grid cells were cross-classified by the categorical variables (zone, prey biomass or percentage prey) in contingency tables. The chi-square statistic was used to compare observed and expected frequencies in the categories [34] and some of the categories were combined for analysis. Two small zones, the Tuli Conservation Zone and Tuli Farms, were excluded from this analysis.

The family of chi square tests conducted were for the following H_0_: 1) the distribution of large prey biomass is independent of the land use zone, 2) the distribution of small prey biomass is independent of the land use zone, 3) the presence or absence of large livestock are independent of the land use zone, 4) the presence or absence of small livestock are independent of the land use zone, 5) the distribution of percentage large prey is independent of the land use zone, and 6) the distribution of percentage small prey is independent of the land use zone.

We followed the Sequential Bonferroni procedure for adjusting significance levels to control Type I error rates for a family of tests and used standardised residuals to interpret lack of independence in contingency tables [34]. Maps depicting the geographic distribution of the classified variables were compiled in ArcGIS 9.3.

## Results

In the family of tests all six null hypotheses were rejected and these six variables were not independent from the zone (Table 3). The assumptions for the chi square statistic are the independent classification of observations and the expected frequency should not be less than five in more than 20% of the categories [34]. We complied with both assumptions after combining some categories for analysis and excluding two small zones.

**Table 3:** Calculated chi square value, degrees of freedom (df), adjusted α value and the chi square test statistic value for the six contingency tables.

Variable	Chi square	df	Adjusted α value (α = 0.05*)	Chi square test statistic	Result
Small livestock	437	7	0.008	19.06	Reject H_0_
% Small prey	460	14	0.010	29.14	Reject H_0_
Small prey biomass	533	21	0.013	37.97	Reject H_0_
Large livestock	639	7	0.017	17.06	Reject H_0_
Large Prey biomass	730	21	0.025	35.48	Reject H_0_
% Large prey	820	14	0.050	23.68	Reject H_0_

*The α value was adjusted for six comparisons following the Sequential Bonferroni procedure [34].

The standardised residuals showed that most large prey biomass occurred in the conservation zones and one was more likely to find marginal to zero large prey in the agricultural zones (Table 4). In contrast, small prey biomass showed no general tendency to be marginal or lower in the agricultural zones compared to the conservation zones, although higher levels occurred in the latter zones. Marginal small prey biomass occurred more than expected in the Central Agricultural Zone, Ngami Agricultural Zone, and Northern Conservation Zone, while medium levels of small prey biomass occurred more than expected in the Southern Conservation Zone, XaiXai Conservation Zone, and Ghanzi Agricultural Zone (Table 5). Standardised residuals for small and large livestock presence were positive for all the agricultural zones and negative for all the conservation zones (Table 6) indicating that most livestock occurred in the agricultural zones. Percentages of large prey biomass were low in agricultural zones where high numbers of cattle occurred and the highest percentage of small prey occurred in the Southern Conservation Zone (Table 7).

**Table 4:** Standardised residuals for large prey biomass (LSU/100 km^2^) categories per Management Zone.

Management Zone	Zero to marginal	Very low	Low to medium	High
Central Agricultural Zone	7.65	−5.35	−7.42	−1.92
Ghanzi Agricultural Zone	0.23	2.21	−1.82	−1.49
Kgalagadi 1 Agricultural Zone	5.07	−2.11	−5.78	−2.40
Kgalagadi 2 Agricultural Zone	0.98	1.47	−2.61	−1.00
Ngami Agricultural Zone	3.66	−2.32	−3.61	−1.32
Northern Conservation Zone	−5.23	−1.14	5.23	12.23
Southern Conservation Zone	−8.47	6.83	9.46	−3.23
XaiXai Conservation Zone	−3.16	0.96	4.90	−1.01

**Table 5:** Standardised residuals for small prey biomass (LSU/100 km^2^) categories per Management Zone.

Management Zone	Marginal	Low	Medium	High
Central Agricultural Zone	8.83	2.03	−5.72	−2.51
Ghanzi Agricultural Zone	−3.59	−3.58	4.85	−0.52
Kgalagadi 1 Agricultural Zone	0.96	2.07	−1.78	−0.67
Kgalagadi 2 Agricultural Zone	−1.58	−0.10	0.84	0.43
Ngami Agricultural Zone	1.32	5.49	−3.86	−2.14
Northern Conservation Zone	1.47	0.27	−4.67	9.58
Southern Conservation Zone	−7.67	−4.25	8.42	−2.32
XaiXai Conservation Zone	−2.45	−2.58	3.86	−1.55

**Table 6:** Standardised residuals for the presence/absence of large livestock (cattle, horses and donkeys) and small livestock (goats and sheep) per Management Zone.

	Large livestock		Small livestock	
Management Zone	Absent	Present	Absent	Present
Central Agricultural Zone	−9.03	6.83	−4.67	5.75
Ghanzi Agricultural Zone	−5.16	3.90	−3.62	4.45
Kgalagadi 1 Agricultural Zone	−7.84	5.93	−6.21	7.65
Kgalagadi 2 Agricultural Zone	−2.60	1.97	−1.77	2.18
Ngami Agricultural Zone	−2.87	2.17	−2.01	2.48
Northern Conservation Zone	9.32	−7.05	5.14	−6.33
Southern Conservation Zone	11.27	−8.53	7.99	−9.83
XaiXai Conservation Zone	2.73	−2.07	1.66	−2.04

**Table 7:** Standardised residuals for the percentage of large - and small prey biomass (LSU/100 km^2^) per Management Zone.

	% large prey			% small prey		
Management Zone	0–5%	5–20%	>20%	0–5%	5–20%	>20%
Central Agricultural Zone	9.53	0.03	−9.59	6.72	0.18	−4.70
Ghanzi Agricultural Zone	4.15	3.07	−5.44	0.29	6.63	−2.59
Kgalagadi 1 Agricultural Zone	7.66	−0.72	−7.41	6.90	2.86	−5.79
Kgalagadi 2 Agricultural Zone	0.95	4.01	−2.61	2.30	−1.02	−1.22
Ngami Agricultural Zone	1.40	2.54	−2.46	3.48	0.64	−2.63
Northern Conservation Zone	−7.69	−2.51	8.77	−5.28	−2.52	4.56
Southern Conservation Zone	−10.99	−2.29	11.98	−9.31	−3.76	7.78
XaiXai Conservation Zone	−2.96	1.45	2.38	−2.55	0.15	1.70

**Figure 2:**
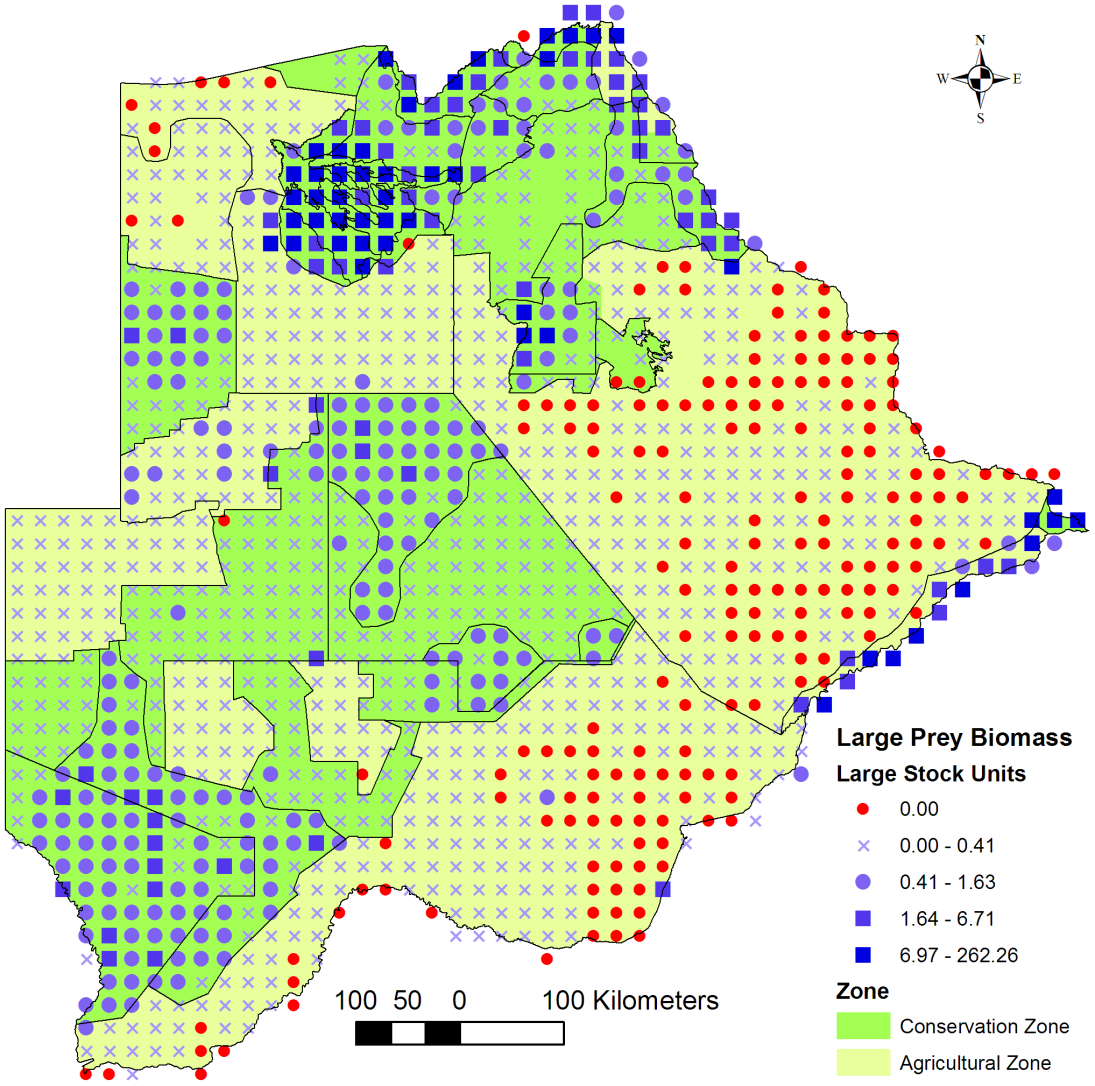
Large prey biomass. Map of the distribution of large prey biomass across the Conservation - and Agricultural Zones. Large prey species refers to wild ungulate species weighing >60 kg and occurring in Botswana that are typical prey for lions (*Panthera leo*), and includes warthog and ostrich but excludes atypical prey such as elephants.

The Northern Conservation Zone consisted of a mosaic of areas with high -, medium - and low large prey biomass (Figure 2). The Okavango Delta had a high biomass along the Kwando/Linyanti/Chobe river system, and also in the eastern part of the Makgadikgadi National Park towards the Boteti River which draws dry season concentrations of zebra and wildebeest to the waterholes [21]. In the drier areas a distribution of a medium biomass seemed to be associated with areas where concentrations of scattered pans occurred and which fell inside the conservation zones. Even though these pans provide water only during the rainy season, the soils surrounding the pans are rich in minerals, produce more nutritious grasses and serve as mineral licks for wildlife [35]. Such areas are found along the north-eastern border with Zimbabwe, and, in the Southern Conservation Zone, the northern part of the Central Kalahari – and the Khutse Game Reserves, and the ‘Schwelle” in the south-western Kgalagadi WMA. The medium large prey biomass recorded in the Ghanzi Agricultural Zone occurred on the commercial farms that lie between the Central Kalahari Game Reserve and the XaiXai WMA. On these farms, integrated livestock and game farming has expanded and the large prey biomass may currently be higher than recorded during the 2001–2007 aerial surveys. Large prey was also recorded over a wide area outside the conservation zones although the biomass was very low.

**Figure 3:**
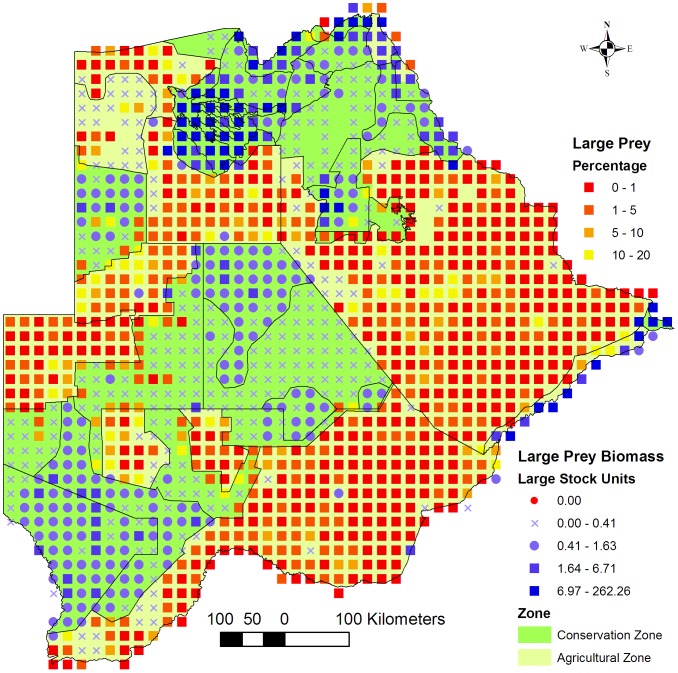
Percentage large prey biomass. Map of the distribution of the percentage large prey biomass across the Conservation - and Agricultural Zones. The percentage was calculated from the total available biomass of large prey and large livestock consisting of cattle (*Bos* spp.), horses (*Equus caballus*) and donkeys (*E. asinus*).

Large prey made up ≤5% of the total large biomass in more than 79% of each agricultural zone (Figure 3). The potential for conflict between people and large carnivores is therefore expected to be high in all the agricultural zones. The exception is the Ngamiland Agricultural Zone (57% of the area) where a large area between the northern Okavango Delta and Namibia is unsuitable for livestock because of the lack of surface water and the presence of the poisonous *Dichapetulum cymosum* plant.

**Figure 4:**
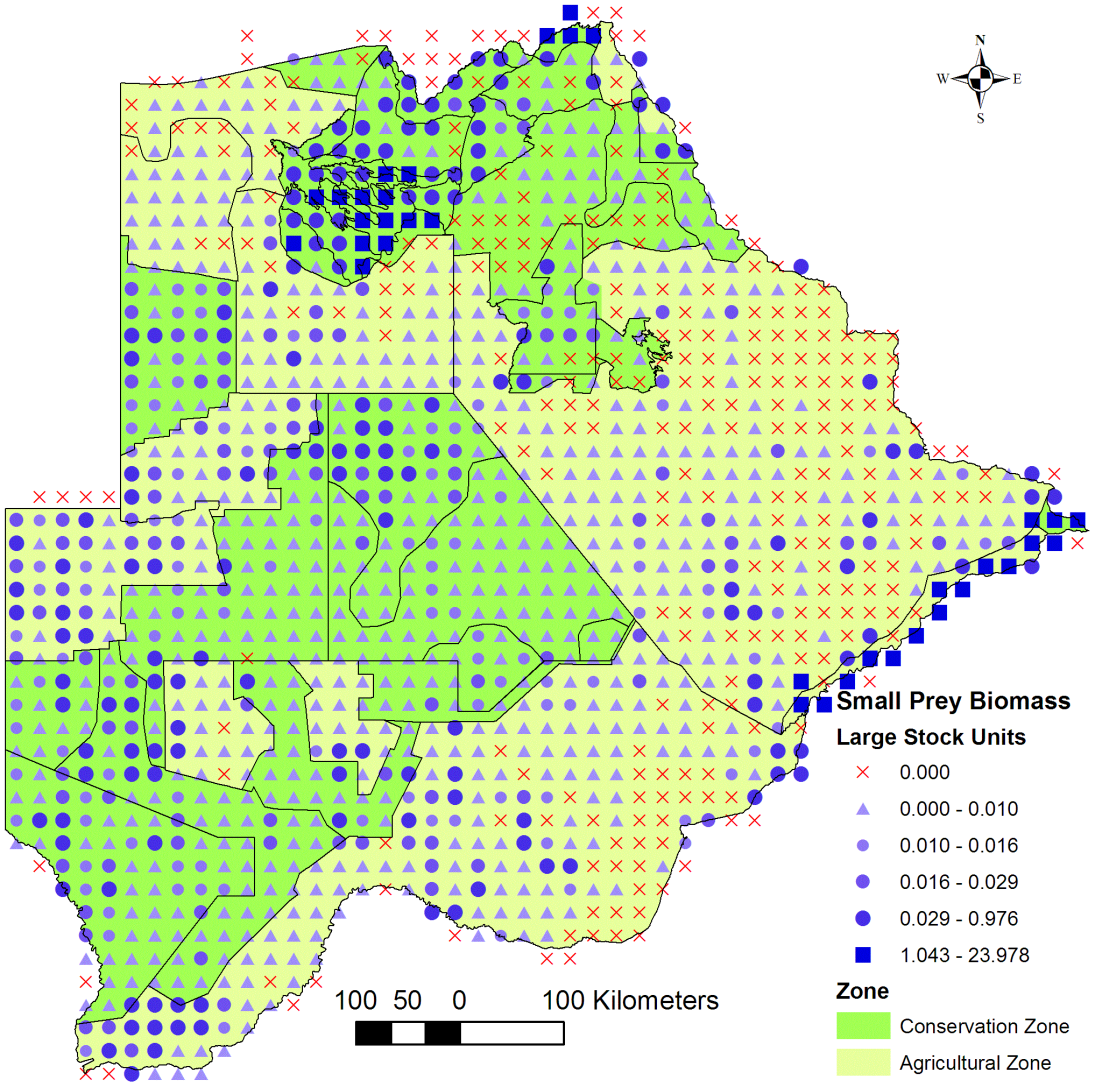
Small prey biomass. Map of the distribution of small prey biomass across the Conservation - and Agricultural Zones. Small prey species refers to wild ungulate species weighing <50 kg and occurring in Botswana that are typical prey for cheetahs (*Acinonyx jubatus*) and African wild dogs (*Lycaon pictus*).

There was a sharp decrease in large prey biomass from conservation - to agricultural areas (Figure 2) which was not observed with the small prey biomass (Figure 4). A medium biomass of small prey was distributed over most of the Southern Conservation -, Ghanzi Agricultural - and XaiXai Conservation Zones with a few localised areas with a high biomass. In the central part of the Dry North Conservation Zone small prey biomass recorded was low to zero in some grid cells. In reality, a high density of small prey, especially duiker (*Sylvicapra grimmia*) and steenbok (*Raphicerus campestris*), was recorded in the area during a ground survey conducted east of Sankuyo Village in 2011 (unpublished data). In aerial surveys a higher error is expected for small ungulates compared to medium-large sized animals because of the former's lower detectability from the air especially in tree-covered areas. This error may be prevalent for the small prey counts across the country.

**Figure 5:**
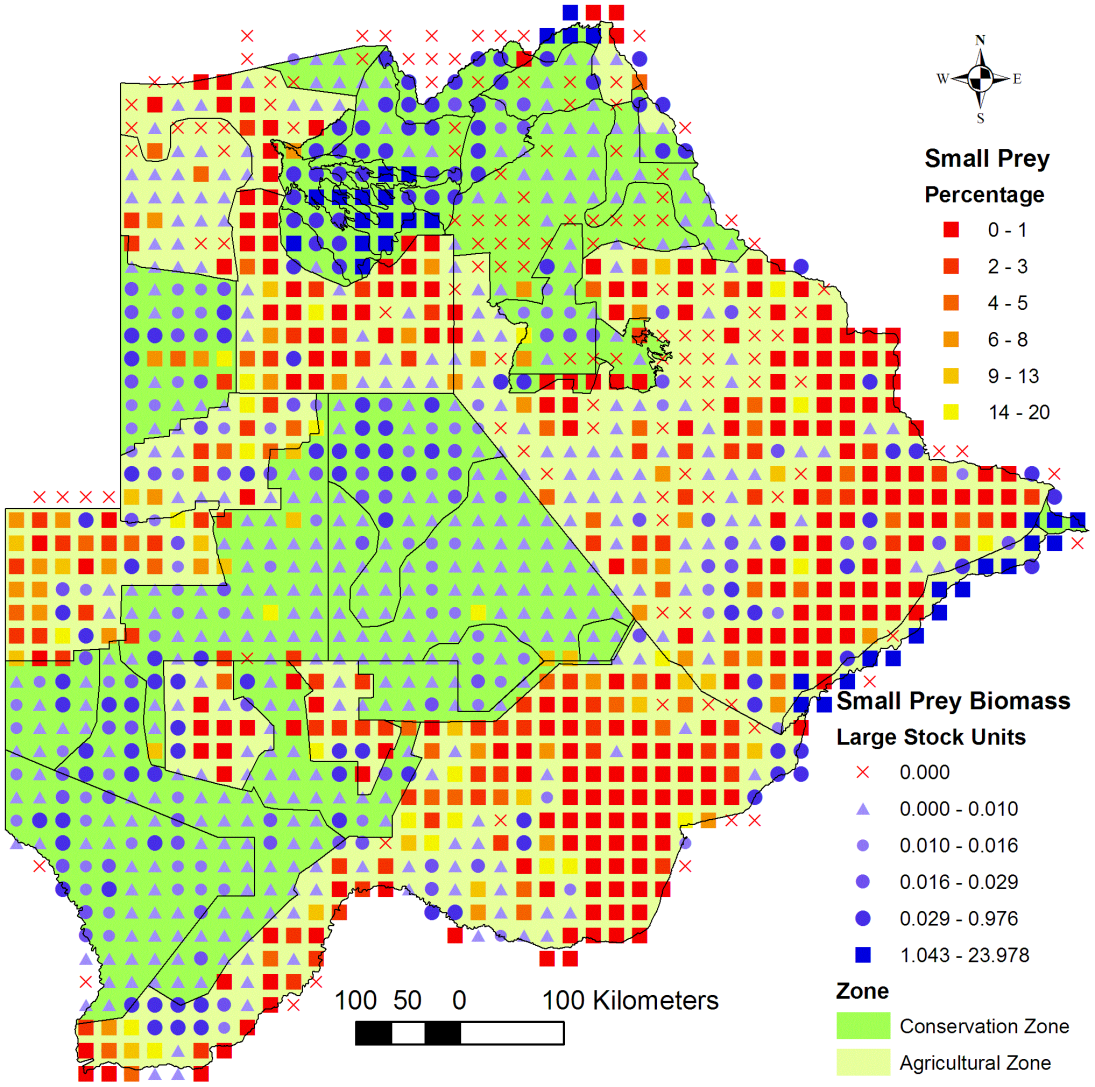
Percentage small prey biomass. Map of the distribution of the percentage small prey biomass across the Conservation - and Agricultural Zones. The percentage was calculated from the total available biomass of small prey and small livestock consisting of sheep (*Ovis* spp.) and goats (*Capra hircus*).

A low (≤5%) percentage small prey, and thus a high potential for conflict between people and large carnivores feeding on small prey, occurred especially along the Panhandle, the western and southern periphery of the Okavango Delta, and in eastern and south-eastern Botswana (Figure 5). In the western and south-western part of the Kgalagadi Agricultural Zone 1 the mixture of areas with medium and low small prey biomass and high percentages of small prey indicate potentially low human-wildlife conflict with large carnivores.

The small Tuli Conservation Zone falls in a low rainfall area and prey biomass was medium to low. The high prey biomass in the Tuli Farms occurs on small fenced game farms. The commercial value of wild ungulates results in game farmers generally having less tolerance for large carnivores than livestock farmers [36] and large carnivores have largely been exterminated on these farms.

## Discussion

We found a clear distinction between the density distribution of large and small prey for large carnivores in Botswana. Since a highly significant, positive linear relationship exist between carnivore density and the biomass of their preferred prey or prey weight range [37] the distinct distribution of large and small prey necessitates focus area conservation strategies for the different large carnivore species.

Large wild prey were primarily limited to the conservation zones, and occurred in the agricultural zones at densities unlikely to sustain large carnivores dependent on large prey. This distribution can be attributed primarily to human disturbance [17], [19], [35] in the form of limited natural resource availability (food and water) for wild ungulates as a result of competition with livestock. This in turn leads to limited wild prey availability, and ultimately to human retaliation against large carnivores for livestock depredations. Cattle are able to competitively displace both wild grazers and mixed-feeders, and the strength of this competition is influenced by seasonal climate variations and habitats [38]. In Botswana, cattle are the predominant livestock type [23] and are widely distributed across the agricultural zones. In the fragile Kalahari sandveld where rainfall is patchy and unpredictable, heavy grazing by cattle has led to bush encroachment, the growth of unpalatable grasses, and expansion of bare soil areas [39], [40]; conditions which are wide-spread across Botswana [41].

In contrast, small wild ungulates are generally less affected by human disturbance [42]. A high density of springbok occur in the southern and western agricultural zones areas presumably because, during the dry season, they could feed on the short green sprouts left by cattle [20]. In the Ghanzi Farms community area steenbok en duiker density ranged between 0.261–4.319 animals/100 km^2^ in spite of the relatively high biomass of cattle and very high small livestock biomass (exceeds that of small prey by a factor of 4) (unpublished data). The widespread, predominantly medium biomass of small wild prey thus affords large parts of the agricultural zones a high conservation potential for large carnivores, effectively increasing the area suitable for their conservation, especially in south-western Botswana. In order to realise this potential, however, legal protection in conjunction with the implementation of realistic site- and species specific human-carnivore conflict mitigation strategies are vital [1].

The distribution of large and small wild prey in the different zones has distinct implications for conflict between people with livestock and the different large carnivores. Lions prefer large prey [2], and as they encounter mainly livestock in the agricultural areas they are the main predator of adult cattle [30], [43]. Because lions pose a serious threat to human lives and most often kill large livestock, conflict with people are ubiquitously fatal [44]. Lions therefore have a very low ecological resilience to human–dominated landscapes and are the least likely large African carnivore to persist in viable populations outside of conservation areas [45]. Lion conflict across the country is expected to follow the same pattern found elsewhere [30], [43], [46], with lion predation on livestock decreasing with increasing distance from the nearest reserve, localized conflict hot spots close to reserve boundaries, and seasonal changes in predation frequency [47]–[50] suggesting that lions are most often transient in human-dominated landscapes. In Botswana, as elsewhere in Africa [45], the survival of lions is bound to the conservation zones, and conflict mitigation efforts focused on conservation area boundaries are crucial to minimize potentially negative impacts on core populations inside. There is some evidence of an edge effect on the Khutse Game Reserve boundary which may eventually threaten the long-term survival of the lion population inside the reserve [51]. Leopards, cheetahs, African wild dogs and spotted hyaenas, on the other hand, prefer small to medium sized prey [2], and their conflict with humans is widely distributed across the agricultural zones with varying intensities between locations [43], [46], [52], [53]. This means more widespread conflict mitigation efforts are needed which focus on conflict hot spots in critical areas.

A number of studies have found that large carnivores prefer wild prey to livestock even when livestock is more abundant [44], [54]–[56]. Conflict reports from the Botswana Department of Wildlife and National Parks show African wild dogs occur widespread across the agricultural zones and it houses almost half of the country's cheetah population [52]. This shows the suitability and importance of the agricultural zones as supplementary conservation areas for these two species, as well as the dedication of institutions to address conflict with humans in different parts of the country. Farmers in Botswana heavily persecute both cheetahs and African wild dogs [46], [57]. Livestock depredation by cheetahs and African wild dogs can be significantly reduced by appropriate husbandry practices such as using herders, guarding dogs or donkeys, and kraaling animals at night [58], [59]. It is thus possible to maintain key areas in the agricultural zones for the conservation of these two carnivore species. However, although the cheetah population trend is thought to be increasing [8], local extinction risks of cheetahs and African wild dogs are high and conflict mitigation remains the most vital and on-going effort for their longterm conservation.

Interactions between large carnivores and their prey are an important part of biodiversity, and conserving an intact carnivore guild thus has a higher priority than single species conservation [60]. In Botswana, the large size, and diverse vegetation and wildlife in the Northern Conservation Zone create an ecosystem ideal for the continued existence of its large carnivore guild. The WMAs surrounding or bordering the protected areas contributes significantly to the protection of the core populations against potential edge effects; it enlarges the conservation area and moves the human-carnivore conflict interface away from protected area boundaries. The Northern Conservation Zone forms part of the Okavango-Hwange lion stronghold [9] and supports the country's main spotted hyaena population [53]. In addition, the mosaic of high to low densities of large prey biomass, and thus dominant competitors, create vital competition refuges for subordinate competitors. Just over half of Botswana's African wild dog population (866 animals) is found in the Northern Conservation Zone [53]. To conserve the intact large carnivore guild in the Northern Conservation Zone effectively, a habitat- and species-level conservation approach is necessary. The integrity of especially the low-density areas need to be maintained, and the uncontrolled development of artificial water points may attract higher densities of large ungulates leading to a corresponding increase in lions and spotted hyaenas numbers, which will reduce refuge areas for subordinate competitors. In addition, non-lethal conflict mitigation strategies around the edge of the Northern Conservation Zone for cheetahs and African wild dogs that cross into the agricultural areas are necessary.

The Southern Conservation Zone is ideal for the conservation of subordinate competitors. The lack of high large prey biomass areas in the Southern Conservation Zone means lions and spotted hyaenas are unlikely to reach high densities, and their numbers are generally kept very low in the agricultural areas through their conflict with humans. The majority of Botswana's cheetahs are found in the Southern Conservation - and its agricultural zones, and almost 90% of its brown hyaena population occurs in the Southern Conservation Zone [53].

The agricultural zones also play a vital role in connectivity by creating corridors between the protected areas in the country, and between transboundary large carnivore populations. Botswana provides the major connecting range for both the largest transboundary African wild dog population and the largest known resident population of cheetahs in southern Africa [10] and maintaining functional linkages between the populations in Botswana and those in its neighbouring countries are vital. In the Southern Conservation Zone, the construction of the Trans-Kalahari Highway and provision of boreholes have led to increasing human settlements and expansion of livestock. In addition, the proposed land use change from wildlife management to agriculture is threatening the connectivity between the Central Kalahari Game Reserve and the Kgalagadi Transfrontier Park via the western Kgalagadi Conservation Corridor. This key wildlife habitat is crucial to the survival of the Kalahari's ungulates [61].

## Conclusion

Our data shows that three conservation strategies are vital in Botswana: maintain the integrity of the conservation zones, maintain high densities of small prey in the conservation and agricultural zones, and successfully mitigate human-carnivore conflict in specific areas. In some parts of Botswana, decreasing wild prey numbers over recent years have had negative consequences for some large carnivore species. One example is the direct link found between decreasing prey availability over the past twenty years in the south-eastern Okavango Delta and a significant reduction in body size of its African wild dogs, a factor that may negatively affect their population viability in the longterm [62]. With regards to conflict mitigation, addressing livestock predation is most pertinent for the conservation of large carnivores, not the least because other mitigation tools, such as translocation [30], [63] and lethal control [64] of stock-raiding large carnivores evidently neither reduce livestock losses nor conserve the large carnivore population involved. Conflict strategies should be ecologically, socially and economically sound [1]. In Botswana, the rural economy is mainly based on cattle which also have an important social value [25], [26]. A key conservation priority is therefore addressing cattle depredation by large carnivores, especially lions, and the resulting retaliation by people. Efforts to force the preservation of large carnivores that pose a threat to human lives and livelihoods in areas with little prey may lead to illegal killing of these animals, and also when poison is used it can have grave consequences for other species. It may also undermine conservation efforts aimed at more vulnerable large carnivore species. To be successful, conservation management strategies must be site- and species specific, pragmatic and maintained in the long term. When the aim is to conserve a large carnivore guild, a wider range of conflict mitigation strategies are required compared to conserving any single species. Importantly, conservation management should never focus on one species to the detriment of another.

## Supporting Information

Spreadsheet S1 **Biomass (LSU/100 km^2^) analyses from the individual aerial surveys.** Large Stock Unit (LSU) conversions of the different wildlife species recorded during the aerial surveys, the biomass (LSU) calculated for the large carnivore prey species occurring in Botswana per aerial survey grid cell, and percentage prey biomass calculated in the different management zones and strata.
(XLS)
